# Indoor Scene Recognition Mechanism Based on Direction-Driven Convolutional Neural Networks

**DOI:** 10.3390/s23125672

**Published:** 2023-06-17

**Authors:** Andrea Daou, Jean-Baptiste Pothin, Paul Honeine, Abdelaziz Bensrhair

**Affiliations:** 1 Univ Rouen Normandie, INSA Rouen Normandie, Université Le Havre Normandie, Normandie Univ, LITIS UR 4108, F-76000 Rouen, France; paul.honeine@univ-rouen.fr (P.H.); abdelaziz.bensrhair@insa-rouen.fr (A.B.); 2 Department of Research and Development, DATAHERTZ, 10000 Troyes, France; jean-baptiste.pothin@datahertz.fr

**Keywords:** scene recognition, indoor localization, deep learning, smartphone sensors, magnetic heading, direction-driven, CNNs, mobile computation offloading

## Abstract

Indoor location-based services constitute an important part of our daily lives, providing position and direction information about people or objects in indoor spaces. These systems can be useful in security and monitoring applications that target specific areas such as rooms. Vision-based scene recognition is the task of accurately identifying a room category from a given image. Despite years of research in this field, scene recognition remains an open problem due to the different and complex places in the real world. Indoor environments are relatively complicated because of layout variability, object and decoration complexity, and multiscale and viewpoint changes. In this paper, we propose a room-level indoor localization system based on deep learning and built-in smartphone sensors combining visual information with smartphone magnetic heading. The user can be room-level localized while simply capturing an image with a smartphone. The presented indoor scene recognition system is based on direction-driven convolutional neural networks (CNNs) and therefore contains multiple CNNs, each tailored for a particular range of indoor orientations. We present particular weighted fusion strategies that improve system performance by properly combining the outputs from different CNN models. To meet users’ needs and overcome smartphone limitations, we propose a hybrid computing strategy based on mobile computation offloading compatible with the proposed system architecture. The implementation of the scene recognition system is split between the user’s smartphone and a server, which aids in meeting the computational requirements of CNNs. Several experimental analysis were conducted, including to assess performance and provide a stability analysis. The results obtained on a real dataset show the relevance of the proposed approach for localization, as well as the interest in model partitioning in hybrid mobile computation offloading. Our extensive evaluation demonstrates an increase in accuracy compared to traditional CNN scene recognition, indicating the effectiveness and robustness of our approach.

## 1. Introduction

People all over the world are increasingly interested in localization and positioning services. Location data can be used for various purposes, including navigation, monitoring, tracking, information services, etc. Several techniques based on different technologies are available to provide an accurate positioning solution in outdoor and indoor environments [1], while global positioning systems (GPS) [2] and point of interest (POI) data [3] have been widely used for outdoor localization, wireless technologies such as WiFi [4], Bluetooth [5], RFID tags [6], and sensor fusion techniques [7] have been popular approaches for indoor localization. Among indoor solutions, vision-based techniques are of great interest because they do not require implementation and maintenance of infrastructure, unlike other indoor technologies.

Vision-based scene recognition can be described as a particular approach to room identification that involves classifying a scene query image to one many scene categories. Traditional vision-based methods for scene recognition mainly focus on images features, which include the image’s global content, objects, and layout visual comprehension. The scene recognition system must therefore have a thorough understanding of the scenes that we encounter in daily life, including both indoor and outdoor environments, in order to be able to assign the appropriate scene category to the given query image. For more than a decade, scene classification has been an active research area benefiting a wide range of applications, such as image retrieval [8], service robots [9], video surveillance [10], augmented reality [11], etc. Indoor scenes are complex because of the diversity of objects and layouts, as well as the variability of lighting and viewing orientations. Therefore, achieving great indoor scene recognition is quite challenging.

Unlike outdoor space, a room-level location in which different rooms within a building are distinguished may be sufficient for most indoor location-based services. The purpose of this paper is to develop a room-level indoor localization system mainly for people who need positioning solutions that are less susceptible to conditions that alter the waves in indoor areas, focusing on easy system installation and usage. Since applications that aid humans in understanding their surroundings are supported by indoor scene recognition systems, it is crucial to develop robust and trustworthy indoor scene classification models. One useful source used for indoor localization is image analysis and classification [12].

Thanks to the success of convolutional neural networks (CNNs), starting with AlexNet in object classification with the large-scale ImageNet dataset [13], research focus on scene classification has been diverted from handcrafted feature extraction methods to deep learning (DL) [14,15,16]. However, one significant drawback of CNNs is the requirement for a large-scale labeled dataset for training, which is not feasible in many applications, such as indoor scene recognition. By applying transfer learning, CNNs pretrained on large-scale datasets (such as ImageNet) are fine-tuned with target scene datasets by making the last layers more data-specific [16].

With recent progress in computer vision techniques, visual place recognition (VPR) can now be considered a promising room-level localization solution. Indoor scene recognition approaches based on CNNs have led to good results in some situations and environments; however, there is still room for improvement. It is therefore necessary to combine other sources of information to overcome the complexity problem. Global positioning systems represent a well-known technology used for outdoor localization but cannot provide accurate indoor positioning due to low signal strength and reduced accuracy in closed and congested environments. On the other hand, WiFi, Bluetooth, and RFID can provide such information, but the price to pay is the installation and maintenance of infrastructure, as well as high sensitivity to indoor conditions (e.g., walls and furniture) [17,18].

In this paper, we propose an approach that takes advantage of smartphone sensors combined with CNNs for indoor room-level positioning. To be located, the user takes a picture of the room scene with their smartphone. Smartphones are easily accessible devices with built-in cameras that are used on a daily basis. These devices are not only endowed with cameras but also equipped with several built-in sensors that provide the opportunity to acquire additional information and therefore build reliable systems for indoor scene recognition [19]. Almost every smartphone has a built-in magnetometer that provides the direction the user is facing, which is known as the magnetic heading (Magnetic heading represents a device’s direction relative to the magnetic north. In general, compass heading is the heading measured clockwise from the magnetic north varying from 0${}^{\circ }$ (north) to 360${}^{\circ }$).

This work proposes a direction-driven multi-CNN indoor scene classification system based on a combination of image features and the magnetic heading from a smartphone camera (i.e., pointing direction). We assume that this additional information can be very informative, given that indoor scene recognition constitutes a complex task in computer vision. The proposed system contains four CNNs, each specific to a definite direction range. Given a query image, the system selects the corresponding CNNs for image classification depending on the magnetic heading of the user’s smartphone camera. In the training phase, we propose overlapping direction ranges, which means that for a given image direction, the image is fed to two CNNs. This allows for more training images per CNN, since two CNNs may share a subset of the training dataset. In the inference phase, two CNNs are involved in query image classification depending on the magnetic heading of the smartphone camera to obtain more comprehensive features. At the end of this process, to further upgrade our model performance, a weighted fusion method is adopted to determine the final image category and predict the user’s specific room location in the indoor space.

The objective of this paper is to present a new approach for room-level indoor localization. Our contributions can be summarized as follows:

1. A novel direction-driven architecture of CNNs is introduced to provide an improvement in indoor scene recognition accuracy. Off-the-shelf pretrained CNNs have predefined architectures, with a fixed input size, which limits additional the information to be provided as an entry. We propose an image classification system guided by supplementary information. The magnetic heading direction of the smartphone assists in vision-based indoor scene recognition, helping the system to identify different specific indoor rooms, taking into account multiple viewpoints.
2. A hybrid computing approach is proposed to address latency, scalability, and privacy challenges. In general, meeting the computational requirements of DL with the limited resources of handheld devices is not feasible. Several works have combined on-device computing with edge computing and/or cloud computing, resulting in hybrid architectures [20]. We take advantage of these new computing techniques to propose a global system computing strategy that meets users’ needs.
3. While several indoor and/or outdoor localization datasets exist in the literature, none of them integrates information other than images. To overcome this issue, we provide a dataset containing images with their respective magnetic heading direction in the metadata.

We conducted experiments in five different indoor scenes and evaluated classification performance according to accuracy on the whole test set. Compared to the scene recognition method based solely on image features, which is a single-CNN-based classification system fine-tuned on an image training set, the proposed model enables significant improvement in recognition accuracy.

The remainder of this paper is organized as follows. Section 2 introduces CNNs, VPR, and the use of magnetic field in localization, along with the advantages and disadvantages of the current computing approaches. Section 3 describes the proposed indoor scene recognition approach, as well as each component of its architecture. Section 4 discusses the partitioning of the proposed DL model. Section 5 depicts the different experiments conducted on a real dataset, in addition to the effect of model partitioning on system computation during inference. Finally, Section 6 concludes the paper with a discussion of future work.

## 2. Background

This section provides a brief discussion of CNNs and their applications in indoor scene recognition, as well as the utilization of magnetic field in localization. Furthermore, we delve into the advantages and disadvantages of current computing approaches.

### 2.1. Convolutional Neural Networks with Transfer Learning

In recent years, CNNs have become a very popular method for image classification and are therefore used in many applications due to their powerful feature extraction ability that allows them to outperform traditional approaches [21]. A CNN is a hierarchical network composed mainly of convolutional layers, activation functions such as rectified linear units (ReLUs), pooling layers, and fully connected layers. The intermediate convolutional layers carry the important responsibility of feature extraction. CNNs can outperform handcrafted feature extraction methods, improving state-of-the-art recognition results [22]. To this end, deep CNN models are necessary, requiring large-scale training datasets to properly estimate the underlying weights. Two research directions have been undertaken to address issues, such as insufficient training datasets and the requirement for lighter architectures, as in the present work.

**Figure 1:**
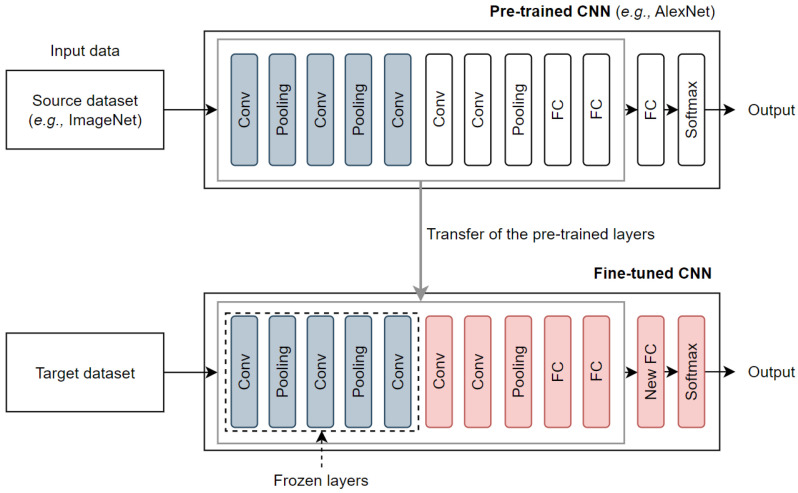
CNN training on a target dataset using transfer learning.

With the evolution of DL, transfer learning has become a popular approach to solve new classification tasks with insufficient training datasets by fine-tuning pretrained CNNs [23]. For example, a CNN model pretrained on large-scale datasets such as ImageNet [13] can be fine-tuned with a training dataset containing images representing the target task. Thus, the CNN weights are updated in an end-to-end manner in the training phase. Freezing the first layers refers to the process of fixing the weights and parameters of specific layers in a pretrained CNN while training on a new task during transfer learning, as shown in Figure 1. This process helps to preserve learned features from the source task that may be useful for the target task and reduces the number of trainable parameters in the network, which can significantly accelerate training and prevent overfitting. In this paper, we use transfer learning to benefit from the generalization and feature extraction capabilities of these pretrained models, which eliminates the need for training from scratch and improves the efficiency of our system.

### 2.2. Lightweight Convolutional Neural Networks

While it is known that the deeper the CNN model, the better the classification performance, light (pretrained) CNN architectures are able to perform well with fewer layers and weights. Light CNN models are intended for resource-constrained environments with low memory requirements for hardware circumstances and good performance for a variety of tasks, balancing between accuracy and efficiency. Examples of such light CNN architectures are SqueezeNet [24], ShuffleNet [25], and MobileNet [26,27].

SqueezeNet [24] is a lightweight CNN architecture that uses 50× fewer parameters than AlexNet [13] while achieving the same accuracy. SqueezeNet enhances accuracy while limiting the parameter count by adopting strategies such as replacing 3 × 3 filters with 1 × 1 filters, reducing input channels, and downsampling late in the network. SqueezeNet is a compact and efficient solution suitable for a variety of deployment scenarios when combined with model compression techniques. ShuffleNet [25] is another small CNN architecture that uses an innovative technique known as channel shuffling to reduce computational complexity and the memory footprint. This approach enables effective information exchange across different network channels. ShuffleNet uses group convolutions and point-wise group convolutions to find a balance between model size and accuracy. MobileNet-v2 [27] is a version of the original MobileNet-v1 [26] built specifically for mobile and embedded vision applications. It uses inverted residual blocks and linear bottlenecks to improve both efficiency and performance. MobileNet-v2 employs depth-wise separable convolutions, which divide the typical convolution into distinct depth-wise and point-wise convolutions, reducing the amount of parameters and operations. It also uses shortcut and residual connections to improve gradient flow and feature reuse during training.

### 2.3. Visual Place Recognition

**Figure 2:**
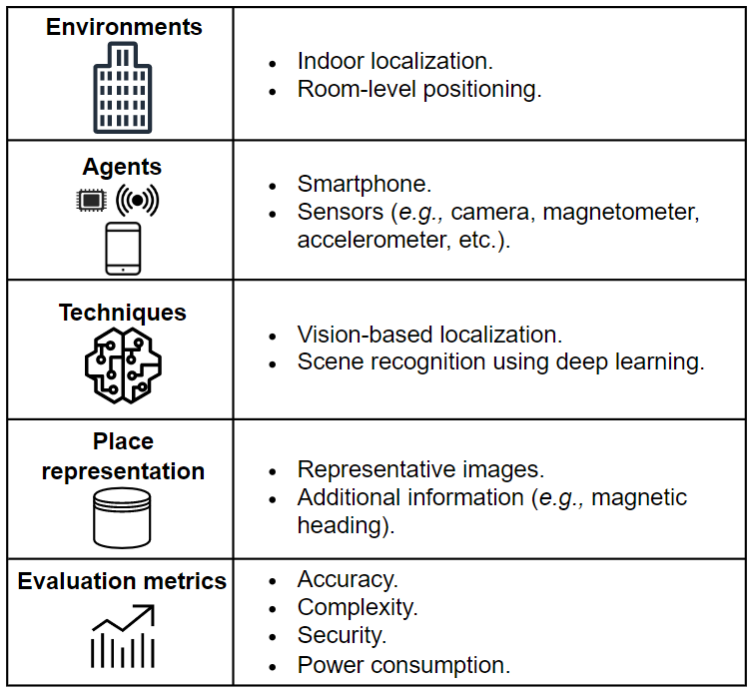
Elements of a visual place recognition (VPR) solution.

Indoor scene recognition is a key point in vision-based indoor localization, namely visual place recognition (VPR). Over the last two decades, image feature detection has been driven by local low-level feature extraction approaches, such as scale-invariant feature transform (SIFT) [28] and speeded-up robust features (SURF) [29], especially in scene recognition [30]. To improve the performance of scene classification systems, researchers have been interested in replacing these traditional feature detection methods with deep neural networks such as CNNs. CNN-based approaches predict the probability of scene categories directly from the entire scene image. VPR systems, which are capable of recognizing one’s location based on an observation, are characterized by several elements [31], as shown in Figure 2.

There are many different types of indoor scene images, including scenes of houses, offices, hospitals, and classrooms. Researchers have been working on diversifying datasets to make classification algorithms more effective. The majority of the available scene datasets include images of external and internal environments, such as MIT Indoor67 [32], Scene15 [33], SUN [34], SUN Attribute [35], and Places [14]. These datasets do not account for changes in rotation, luminance variation, or point of view. As the common well-known datasets from the computer vision community cannot be used in practice, variations of practical experiments in real-world case scenarios led to the creation of datasets based on available information [36]. As indoor scene recognition is a key component of vision-based indoor localization, the created datasets directly support real-world applications.

### 2.4. Magnetic Field in Localization Applications

Magnetic field is used for localization and tracking in a variety of applications [37,38]. Integration of magnetic field data as fingerprints, along with visual information for indoor localization, was studied in [39,40]. Both papers cited above present solutions for indoor localization based on magnetic field data as fingerprints and visual information from a smartphone camera, as well as DL approaches. Our proposed work aligns with the concept of fusion of magnetic and visual sensor data, but we propose using built-in smartphone sensors to provide valuable information about the device’s magnetic heading with respect to magnetic north.

Smartphone compass sensors capture magnetic fields external to the device and are linked to the device’s accelerometer to determine its position. In contrast to a traditional compass, which must be laid flat, a smartphone can be used in any position. As explained in Section 1, we used the magnetometer and accelerometer of a smartphone for magnetic heading estimation. The information about the gravity vector must be integrated based on the accelerometer. On the other hand, the magnetometer provides the sensor heading (i.e., orientation around the gravity vector), which is information that the accelerometer alone cannot provide [41]. This information is then integrated for the estimation of the magnetic heading in the vertical portrait position of the smartphone, which allows for correct acquisition of the heading of the camera when taking an image.

Smartphone-based sensors are an important component in the mobile computing domain, serving as a platform for new applications. However, the accuracy of these sensors is critical for such applications. A smartphone magnetometer is a Hall effect sensor that perceives magnetic fields in an active manner [42]. Artificial and natural magnetic fields are numerous and variable. Thus, the compass sensors of smartphones must be recalibrated on a regular basis in order to reanalyze the present magnetic fields and determine where the north is. Estimation of the magnetic heading is primarily dependent on:

- The device: different sensors have varying precision, sensitivity, and stability. Various built-in sensors and algorithms utilized by smartphone manufacturers lead to different magnetic field measurements.
- The user’s surroundings: other electronic devices commonly found inside buildings cause interference and magnetic perturbation. The omnipresent magnetic field is disrupted by ferromagnetic materials used in buildings, affecting magnetic field measurements and causing inaccurate direction and position information.

All the above challenges may affect the performance of localization systems relying on magnetic field data.

### 2.5. Computing Strategies

Smartphones are widely used and easily accessible devices. With regard to processing power, memory size, and battery life, these devices still have limited resources. Smartphone hardware technology has improved to the point where it can now handle some difficult calculations but not enough to support computationally demanding tasks such as decision making and image recognition. Additionally, these heavy tasks consume more battery power, making them power-hungry. Thus, a solution is needed to overcome these limitations.

**Figure 3:**
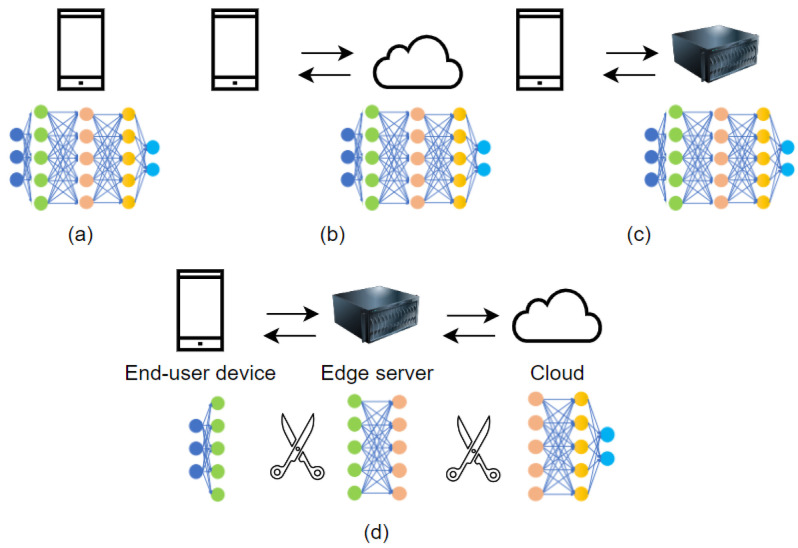
(**a**) On-device computation. (**b**) Cloud-based computation. (**c**) Edge-server-based computation. (**d**) Hybrid computation.

Different computing strategies for inference have been proposed to overcome application constraints. As shown in Figure 3, there are four common computing approaches: (a) on-device computation, (b) cloud-based computation, (c) edge server-based computation, and (d) hybrid computation.

**On-device computation** is a strategy in which computational operations are carried out directly on end-user devices (e.g., IoT devices, smartphones, and tablets). As smartphones are equipped with cameras and other useful sensors, they enable the design and implementation of many beneficial applications. Unfortunately, some applications are computationally demanding, limiting their use on smartphones, owing to limited processing and computation power, limited battery life, and insufficient memory and storage capacity.

To overcome these limitations, the recently introduced mobile computation offloading approach is a potential solution, as it helps in offloading computation (i.e., full offloading) or parts of computation tasks (i.e., partial offloading) from mobile devices to remote cloud servers, local edge servers, or both (i.e., edge–cloud computing). Computation offloading involves the transfer of computational tasks to a separate processor. As a result, information must be moved from the end-user device, which serves as the data acquisition device, to the server.

**Cloud-based computation** is leveraged for its processing capabilities and memory capacity, as the data are processed on the cloud side, not on limited-resources devices. It also helps to store data if required, which can be accessed later [43]. Moving computation responsibilities and storage operations away from the main processor of a smartphone can help save computation time by lowering the running cost of computation-intensive tasks. Basically, using a server for inference keeps the mobile application simple because all the complex tasks are performed on the server. Thus, when implementing the system in a mobile user-friendly application, smartphones deficiencies do not have to be considered. This allows for unrestricted computing performance and mobility at any moment from any user’s device. However, limited battery energy impacts the use of the smartphone for heavy tasks, as it requires more energy due to high processing requirements, screen use, and continuous data acquisition by sensors. Given the data, the application must perform a series of tasks requiring specific computation to achieve the desired result. Cloud-based computation can help to save energy on mobile devices, as computation-intensive tasks are offloaded to the cloud, and improve reliability by storing and accessing data on the cloud side, which decreases the risk of data loss on mobile devices [43]. In addition to these advantages, there are numerous disadvantages [20]:

- Bandwidth and scalability are main issues in cloud-based computation, that are exacerbated with an increasing number of connected mobile devices and increased data transfer volume. Likewise, as the number of connected devices increases, sending data from mobile devices to the cloud introduces scalability problems, as the cloud entry can become a bottleneck.
- Latency: Cloud-based computation may not always be a suitable solution when working on real-time applications, as data transfer to the cloud may suffer from extra network queuing and transmission delays, leading to high latency.
- Service availability: Due to wireless bandwidth limitations, network disconnection, and signal attenuation, a connection to the cloud might not always be possible. A sudden internet outage stops application functionalities, as cloud-assisted systems rely on the network to transfer data from users’ mobile devices to the cloud server and vice versa.
- Privacy: The data sent from end-user devices to the cloud may contain sensitive information, leading to privacy concerns. Data sharing and storage in the European Union and the European Economic Area must comply with the General Data Protection Regulation (GDPR), an EU regulation on data protection and privacy.

**Edge-server-based computation** can be adopted as a solution to reduce offloading, resulting in communication delays between mobile devices and the cloud. Instead of offloading tasks to a remote cloud, mobile devices can offload tasks to closer edge servers that help meet delay requirements by using short data transfer intervals. Edge computing is an appropriate solution in cases in which the user or the system cannot wait the time it takes to send the data to a large remote center (cloud) and have results sent back. With computing power on the edge side, decisions and results are received quickly. In addition to the power of edge servers, which is greater than that of end-user devices, these servers conserve network bandwidth usage by performing on-site computing and only sending necessary information to off-site servers. Thus, edge-server-based computation helps meet latency, scalability, and privacy requirements by keeping sensitive data close to the source [20]. However, there are differing opinions on the safety of edge computing, while some consider edge servers to be a safe option for protecting sensitive data, others believe that data breaches occur more frequently with edge infrastructure due to inadequate security measures. As a result, a robust edge security system must be installed to protect the edge computing infrastructure and ensure its viability. Using edge computing, less data are sent to the cloud, which may aid in lowering operational costs. On the other hand, the initial investment in hardware and infrastructure for on-premises edge server systems can be considerable. In addition to pricing the server hardware and installing it in a suitable location, infrastructure requires regular maintenance and updating.

**Hybrid computation** refers to a combination of different computing approaches that requires the integration of several resources, such as mobile devices, edge servers, and cloud servers [20]. As previously mentioned, computation offloading can be full or partial. Full offloading means that the application is fully executed on the cloud or edge-server side, whereas partial offloading, which applies to hybrid computing, means that an application is executed on different processing resources. Hybrid computation combines the computational capabilities of mobile devices with the resources of cloud servers and/or edge servers located close to the devices. In general, lightweight tasks or initial processing are performed on mobile devices, while the more computationally intensive parts are offloaded to the cloud and/or edge server for execution. There are various advantages of using hybrid computation:

- Scalability: Cloud and edge servers offer high-performance computing capabilities, which enables the efficient execution of challenging tasks. The ability to scale resources dynamically based on the workload or demand ensures that the computational requirements of the tasks can be met effectively.
- Network bandwidth: Offloading computationally intensive tasks to servers minimizes the quantity of data that needs to be transferred across the network, which is important when bandwidth is limited. The overall network traffic can be reduced by transmitting only the necessary inputs and receiving only the processed results.
- Latency: Determining which tasks to offload to servers is critical to reduce latency. Offloading computationally complex operations that benefit from server-side processing can increase real-time performance and reduce the total response time. Lightweight tasks can be maintained on the mobile device for faster execution.
- Centralized maintenance and updates: When computationally intensive tasks are offloaded to servers, the server infrastructure carries the main responsibility of maintaining and updating the system. This decreases the complexity and effort necessary for maintenance and updating of each mobile device, simplifying overall system management.
- Energy: Hybrid computing architectures can help with energy efficiency. Energy consumption can be lowered by executing lightweight tasks or initial processing on-device. Edge computing lowers the requirement for long-distance data transmission, saving even more energy. Using cloud servers for resource-intensive tasks allows for more efficient server infrastructure use and potentially reduced power consumption.
- Privacy: When employing hybrid computing architectures, privacy is a crucial factor, especially when external servers are involved. To guarantee that privacy requirements are respected, task offloading policies should be carefully considered. Offloading only non-sensitive data to servers while retaining sensitive data on the mobile device can help to preserve user privacy.

## 3. Proposed Approach

In the following, we provide a detailed description of the proposed room-level indoor localization system based on image classification using direction-driven multi-CNNs.

### 3.1. Localization System Architecture

Knowing that indoor scenes are very complex due to strong change in viewpoints and high similarity between scenes, additional information could be of great interest. Our intuition relies on the assumption that the camera heading of the user smartphone relative to magnetic north can be very informative. It informs the image classification system as to which way the smartphone’s camera is facing. We combine accelerometer information with magnetometer data for magnetic heading estimation, which allows for correct orientation relative to north when the smartphone is held vertically (i.e., determining the camera facing when capturing an image).

**Figure 4:**
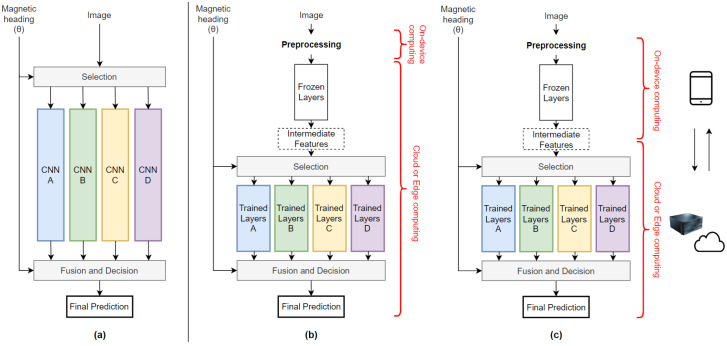
(**a**) Architecture of the proposed system with four CNNs. (**b**) Computing strategy with full offloading, with the four CNNs partitioned in the common submodel (i.e., frozen layers) and the other four submodels (i.e., trained layers *A*, *B*, *C*, and *D*). (**c**) Computing strategy with partial offloading.

**Figure 5:**
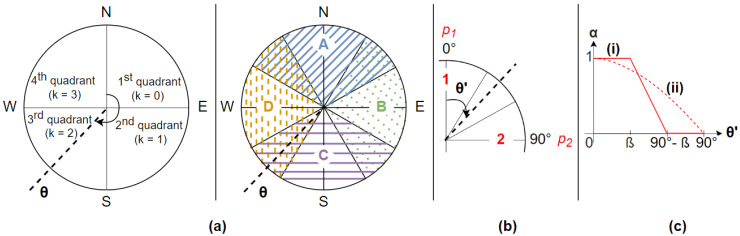
(**a**) CNN selection depending on the magnetic heading of the image (θ). (**b**) Weighted fusion strategy. (**c**) Fusion techniques: (**i**) piecewise linear and (**ii**) cosinusoidal.

We propose a direction-driven multi-CNN system for indoor scene recognition that takes into account the magnetic heading of the user’s smartphone (θ). The global architecture illustrated in Figure 4a consists of three main components: the selection block for direction-driven model selection, the image classification model defining four CNN models, and the fusion and decision block for combination of the obtained results. These three components are described in detail in Section 3.1.1, Section 3.1.2 and Section 3.1.3, respectively. In order to cover the four ranges of orientations (*A*, *B*, *C*, and *D* in Figure 5a), the proposed classification system contains four CNNs, denoted as *A*, *B*, *C*, and *D*. The use of four ranges of orientations is motivated by small datasets and the need to avoid underfitting or overfitting. Dividing the heading directions into more ranges would necessitate a greater number of images for each range to provide adequate representation. This strategy allows us to assign a fair number of images to each range, guaranteeing that the CNNs can efficiently learn distinguishing features. Algorithm 1 presents the process followed for indoor scene image classification in the online phase.
**Algorithm 1:** Inference classification methodology**Input**: Query image, Magnetic heading θDetermine the quadrant to which the magnetic heading θ belongsSelect the two corresponding CNNs according to the value of the parameter *k* as defined in Figure 5a$p_{1}$ = Estimated probabilities with the first selected CNN$p_{2}$ = Estimated probabilities with the second selected CNN$\alpha (\theta^{\prime })$ = Weighting parameter of the fusion method with (3) or (4)$p=\alpha \left(\theta^{\prime }\right)\;p_{1}+\left(1-\alpha (\theta^{\prime })\right)\;p_{2}$Predict the image category with max(p)**Output**: Prediction of the specific indoor room

#### 3.1.1. Selection Block

The main objective of the selection block is to select two of the four available CNNs in order to use them for indoor scene image recognition. The four CNNs of the proposed classification system are trained and validated on specific subsets of data based on the magnetic headings of the collected images. During inference, the two CNNs are selected according to the quadrant to which the magnetic heading (θ) of the camera belongs when the user takes the image. More precisely, according to the *k* parameter defined in Figure 5a, the selection rule is as follows:

- Between north and east (i.e., k=0): select CNN *A* and CNN *B*;
- Between east and south (i.e., k=1): select CNN *B* and CNN *C*;
- Between south and west (i.e., k=2): select CNN *C* and CNN *D*;
- Between west and north (i.e., k=3): select CNN *D* and CNN *A*.

The outputs of the two selected CNNs are subject to weighted fusion performed in the fusion and decision block, as described in Section 3.1.3. First, we provide a detailed description of the image classification models.

#### 3.1.2. Image Classification Models

We propose a generic system that can include all types of CNNs used for image classification. Pretrained CNNs are trained on more than a million images from the ImageNet dataset [13]. Consequently, these networks learn rich feature representations from a wide range of images. Instead of building CNN models from scratch, we investigated mobile-compatible pretrained CNNs, namely SqueezeNet [24], ShuffleNet [25], and MobileNet [26,27]. These light CNN models have demonstrated a good tradeoff between accuracy and efficiency while addressing resource-constrained environments, including low memory and hardware requirements.

By transfer learning, we fine-tuned these pretrained CNN models as follows. The layer directly preceding the classification layer (fully connected layer or convolutional layer) of the pretrained CNN is replaced with a new layer having a number of outputs equal to the number of categories in the target dataset. A softmax activation function is introduced at the output of the CNN with a number of neurons equals the number of categories to obtain a probability vector as an output. A well-known technique in transfer learning consists of freezing some trainable layers. The weights of those frozen layers are not updated during fine tuning. In general, the frozen layers are selected from the first convolutional layers of the model because the last convolutional layers are more data-specific; therefore, applying fine-tuning to these layers is important to enhance learning quality. Moreover, freezing the weights of several layers can significantly speed up network training.

#### 3.1.3. Fusion and Decision Block

As mentioned in Section 3.1.1, two CNNs are selected based on the quadrant to which the magnetic heading (θ) of the image belongs. Therefore, a weighted fusion technique is applied to the two probability vectors ($p_{1}$ and $p_{2}$) corresponding to the inference outputs of the two selected CNNs. The adopted principle in the fusion block is that when classifying an indoor scene query image, each of the two selected paths contributes to the final decision by a factor depending on the value of θ.

In order to provide a single formulation for all four possible quadrants shown in Figure 5a, we represent them in a single quadrant using the modulo operation as follows

(1) $$\theta^{\prime }=\theta \;\mathrm{mod}\;90^{\circ },$$

namely the modified magnetic heading of the smartphone camera ($\theta^{\prime }\in \left[0^{\circ },\;90^{\circ }\right])$. As depicted in Figure 5b, $p_{1}$ corresponds to the probability vector at the output of the specific CNN for the range of $\theta^{\prime }$ centered at the vertical axis and $p_{2}$ at the output of the CNN whose specific range of $\theta^{\prime }$ is centered at the horizontal axis. Thus, the proposed fusion method is defined as

(2) $$p=\alpha \left(\theta^{\prime }\right)\;p_{1}+\left(1-\alpha (\theta^{\prime })\right)\;p_{2},$$

where $\alpha (\theta^{\prime })$ is the weighting parameter calculated to combine the two probability vectors ($p_{1}$ and $p_{2}$) as described in Algorithm 1.

We propose two weighted fusion strategies based on the smartphone’s magnetic heading. The first strategy is piecewise linear weighted fusion, as represented in Figure 5c(i). Inspired by fuzzy logic, let β be the hyperparameter defining the different intervals of weighting that can take values in the range of $\left[0^{\circ },\;90^{\circ }\right]$. For this first proposed fusion method, the weighting parameter ($\alpha (\theta^{\prime })$) is defined as

(3) $$\alpha \left(\theta^{\prime }\right)=\left\{\begin{array}{cc} 1 & \;\mathrm{if}\;\theta^{\prime }\in \left[0^{\circ },\;\beta \right]\\ \frac{1}{2\beta -90^{\circ }}\;\theta^{\prime }+\frac{\beta -90^{\circ }}{2\beta -90^{\circ }} & \;\mathrm{if}\;\theta^{\prime }\in \left[\beta ,\;90^{\circ }-\beta \right]\\ 0 & \;\mathrm{if}\;\theta^{\prime }\in \;\left[90^{\circ }-\beta ,\;90^{\circ }\right] \end{array}\right.$$

with a special case when $\beta =45^{\circ }$. In this case,

(3a) $$\alpha \left(\theta^{\prime }\right)=\frac{1}{2}\;\mathrm{if}\;\theta^{\prime }=\beta$$

In this paper, we treat the following three cases of linear weighted fusion: $\beta =0^{\circ }$, $\beta =30^{\circ }$, and $\beta =45^{\circ }$. The second proposed fusion strategy is cosinusoidal weighted fusion, as illustrated in Figure 5c(ii), with $\alpha (\theta^{\prime })$ defined as

(4) $$\alpha \left(\theta^{\prime }\right)=\cos\left(\theta^{\prime }\right)\;\forall \theta^{\prime }\in \left[0^{\circ },\;90^{\circ }\right]$$

After applying one of the fusion techniques, the category with the maximum classification probability is selected, namely

(5) max(p).

This leads to the final prediction of the specific indoor room.

## 4. Global System Architecture

In this section, we discuss DL task partitioning and propose a hybrid computing approach to address latency, scalability, and privacy issues.

### 4.1. Computing and Partitioning Deep Learning Tasks

DL task inference can be performed on cloud servers, referred to as cloud-based deep inference, or on edge servers, referred to as edge-server-based deep inference. Alternatively, inference can be performed locally using mobile CPU and GPU, referred as on-device deep inference [20].

These tasks are computationally intensive. Even after the creation of light mobile-compatible CNNs, smartphones remain clearly inferior to edge and cloud servers in terms of performance, as several CNNs may be needed during inference in some applications. In the case of image classification, the inference computational demands of CNNs are strongly reliant on the increase in computing power. In general, meeting computational requirements of DL necessitates cloud computing, as it guarantees limitless on-demand processing power.

Recent studies [44,45] have shown that splitting the network between the mobile device and cloud and/or edge servers can improve the end-to-end latency of CNN inference. One way of using hybrid computing with partial offloading with DL models is CNN model partitioning [46,47]. In such approaches, instead of creating an application handling everything, CNN architectures are distributed between the mobile device and cloud and/or edge server, as shown in Figure 3d. Thus, some layers are computed on the mobile device while other layers are computed by the cloud and/or the edge server, which may reduce the computation power required on the smartphone. The key aspect when distributing computing between the mobile device and the cloud and/or edge server is which data must be stored and processed locally. The optimal computation partitions for offloading are difficult to detect, requiring a separate study and analysis.

### 4.2. Partitioning of the Proposed Model

As explained in Section 4.1, DL model partitioning is the process of dividing a DL model into multiple parts that can each be deployed and run on different computing devices and servers. In the proposed indoor scene recognition system, several CNN models need to be saved and used during inference, requiring an increase in memory capacity and computation power. Including all the needed models in the mobile application bundle also significantly increases its download size, up to many megabytes (MB) in practice. The direction-driven CNNs of our proposed model have a common inference part because some of the layers of the CNNs are frozen during the training phase. Unlike trained layers, which are trained and validated on specific subsets of data based on the magnetic headings of the collected images, this part should only be computed once, helping to reduce the inference time and the size of the global DL model (composed of multiple direction-driven CNNs). Thus, the architecture of the proposed system represented in Figure 4a is partitioned into five submodels: the common submodel (i.e., frozen layers) and the other four submodels (i.e., trained layers *A*, *B*, *C*, and *D*).

Two computing strategies are proposed; Figure 4b represents full offloading (i.e., cloud-based computing or edge-server-based computing), while Figure 4c constitutes partial offloading (i.e., hybrid computing). In the case of full offloading, the captured image is preprocessed and sent to the cloud or edge server for full computation. In the case of partial offloading, the common submodel is computed on the user’s end device (making preliminary predictions before sending the data), and the output intermediate features are sent to the server. Then, the other four submodels are computed on a cloud or edge server. For these two computing strategies, the final prediction (i.e., user’s room-level position in the indoor environment) is sent back from the server to the user side.

The primary goal is to minimize the end-to-end latency while respecting end-user devices and server constraints mentioned in the previous subsections. Partitioning into submodels is based on the communication and computational costs of the submodels; thus, it depends mainly on the layer types, the per-layer output size (i.e., activation), the per-layer data communication latency, the per-layer computation latency (i.e., server and end-user devices processing latency), and the memory footprint. As described in the following section, we conducted experiments in order to provide a deep insight into the proposed partitioning of the direction-driven model.

## 5. Experiments and Results

### 5.1. Dataset Preparation

To construct and evaluate the proposed indoor scene classification system, we first created a dataset of images with their respective magnetic headings with respect to magnetic north. The prepared dataset includes informative images of the indoor environment with different perspectives of the studied rooms.

To ensure an efficient data collection process, we designed an Android application that uses the smartphone’s built-in sensors. When capturing images using this application, each image is collected with the corresponding magnetic heading saved in its metadata. For data collection, the smartphone was held in the portrait/vertical position. The RGB images were cropped and saved at a size of 1088 × 1088 pixels to avoid distorting the shapes of the objects in the images when resized. The dataset was prepared using the main rear camera of a Samsung Galaxy A51.

**Figure 6:**
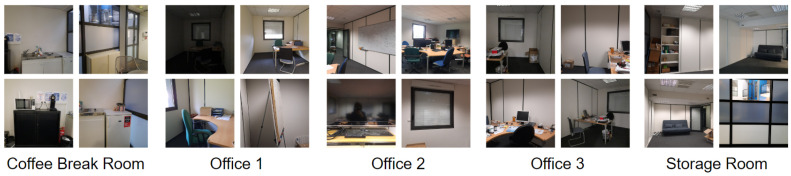
Examples from different classes of the collected dataset.

The indoor environment studied in this work has five rooms: coffee break room, office 1, office 2, office 3, and storage room. To provide diverse and representative data, the data collection process was conducted over several days. We took precautions to maintain consistency during the data collection period. Two data collection rounds were conducted. In the first round, we took eight images per position (i.e., a given standing location) in each room with different orientations. Each position used to collect images results in a distinct perspective. Images were collected at orientations of 0° (North), 45°, 90°, 135°, 180°, 225°, 270°, and 315°. These images were used for offline training of the proposed direction-driven multi-CNN system. In the second round, we took an average of 20 images per position in each room, with different positions than the first round and a full 360-degree rotation in each position to take all the heading viewpoints into consideration. The entire dataset was then cleaned by deleting uninformative images (i.e., images constituted mostly of walls, windows, etc.). We obtained between 100 and 200 images per class depending on the room dimensions and complexity. A part of these collected images was used for training and validation of the system, and the rest were used for assessment of the classification accuracy (50% for the training phase and 50% for the testing phase). Figure 6 shows some examples from the collected dataset.

### 5.2. CNN Training and System Testing

For the proposed indoor scene recognition system based on the direction-driven multi-CNN architecture, the four CNN models need to be trained and validated in order to be implemented. To assess its performance, the proposed classification system was evaluated on the totality of the testing set. We also used the testing set to examine the relevance of the fusion strategies described in Section 3.1.3.

As previously mentioned, since we had few real data points, we relied on CNN models pretrained on ImageNet [13], and fine-tuned them using the real dataset. We examined three well-known mobile-compatible pretrained CNNs: SqueezeNet [24], ShuffleNet [25], and MobileNet version 2 [27]. In order to provide a baseline system, we trained and fine-tuned a single CNN model with the totality of the training and validation sets. In order to provide a fair comparative analysis, we considered the same pretrained CNN used for our proposed recognition system.

Models were optimized using a batch gradient descent optimizer with a learning rate equal to 0.001. Note that pretrained CNNs take fixed image sizes and a defined number of input channels; therefore, all the images in the dataset were preprocessed (Images from our real dataset were scaled to 224 × 224 × 3 to respect the dimensions accepted by the input layer of the pretrained CNNs ShuffleNet and MobileNet and to 227 × 227 × 3 when working with SqueezeNet). We trained the models for a maximum of 500 epochs. In order to avoid overfitting, the CNN training stopped automatically when the validation loss starts began increasing while the training loss was still decreasing. Simulations were implemented using MATLAB R2019a.

### 5.3. Performance Evaluation

We computed the standard performance metric for image classification to assess performance. Test accuracy is defined as

$$\mathrm{Accuracy}=\frac{\mathrm{Total}\;\mathrm{number}\;\mathrm{of}\;\mathrm{test}\;\mathrm{images}\;\mathrm{correctly}\;\mathrm{classified}}{\mathrm{Total}\;\mathrm{number}\;\mathrm{of}\;\mathrm{test}\;\mathrm{images}}.$$

Five Monte Carlo simulations were conducted to evaluate our direction-driven multi-CNN model, as well as the single-CNN baseline system. The average test accuracies, denoted as Accuracy_*avg*_, are presented in Table 1 for the three pretrained CNN models and the fusion strategies. The proposed indoor scene recognition approach outperformed the baseline system in terms of accuracy for all proposed fusion strategies. The results show that the linear weighted fusion with $\beta =0^{\circ }$ achieved the best performance, proving the necessity of combining the two selected CNNs.

Additional tests were carried out to evaluate the performance of various selection rules and their impact on overall system accuracy. We investigated several scenarios, including one in which the system selects the quadrant that is completely opposite to the magnetic heading, resulting in the selection of the two opposite CNNs. For example, if the magnetic heading is between north and east (i.e., k=0), select CNN *C* and CNN *D* (i.e., the complete opposite of the proposed selection block approach, which selects CNN *A* and CNN *B* in this case). We also assessed the system’s performance when only one CNN was chosen randomly from the four available CNNs, as well as when one specific CNN was chosen for all test images. Table 2 shows the results of these tests, which demonstrate how including these alternate scenarios considerably affects the system’s accuracy. The performance in these three scenarios falls significantly short of what the proposed approach presented in Algorithm 1 achieves. In terms of accuracy, the system performs worse than the baseline system when using the opposite two CNNs to the corresponding magnetic heading quadrant for fusion or when omitting the fusion block and instead depending on one of the four available CNNs. These findings highlight the importance and efficacy of the proposed approach, demonstrating higher accuracy. The proposed selection block and fusion block are crucial in improving the system’s performance, resulting in increased accuracy for indoor scene recognition.

### 5.4. Stability Analysis: Effect of Sensor Accuracy on the System

As explained in Section 2.4, there are several challenges that can affect the accurate estimation of the magnetic heading, impacting the overall performance of the proposed localization system. In [48], a sensor accuracy test was conducted in a large industrial hall with seven different mobile devices with non-identical built-in sensors, analyzing the impact of a harsh environment and different hardware on smartphones’ digital compass estimation. At each position in the studied environment, the divergence of the magnetic heading provided by the smartphones from the correct heading from the construction plan was recorded. Examination of the collected device measurements showed that one-half of the overall results were below a magnetic heading error of 20${}^{\circ }$.

To analyze the effect of the error when estimating the magnetic heading on the proposed system’s performance and stability, we conducted magnetic heading error simulations. We assumed that the magnetic heading error, denoted as *e*, follows a normal distribution:

(6) $$e∼N(\mu ,\sigma^{2}),$$

Thus, the normal probability density is guided by the mean (μ) and the standard deviation (σ) and is defined as follows:

(7) $$P\left(e_{i}\right)=\frac{1}{\sqrt{2\pi \sigma^{2}}}\exp\left(-\frac{{\left(e_{i}-\mu \right)}^{2}}{2\sigma^{2}}\right).$$

We computed the commonly used image classification performance measure to assess the stability of the proposed system. This time, the accuracy can be measured as

(8) $$\theta_{e}=\left(\theta +e\right)\;\mathrm{mod}\;360^{\circ },$$

where θ is the magnetic heading of the smartphone’s camera when the image is captured, and *e* is the random Gaussian error as previously defined. The expression of the modified magnetic heading (1) leads to

(9) $$\theta^{\prime }=\theta_{e}\;\mathrm{mod}\;90^{\circ }.$$

Based on [48], knowing that we are not working in an industrial indoor environment and that smartphones have come a long way over the past few years, we simulated error values following a normal distribution function with several values of σ and $\mu =0^{\circ }$. The obtained results are represented in Table 3. The results show a performance reduction when simulating an error following a normal distribution with $\mu =0^{\circ }$ and $\sigma =\{30^{\circ },60^{\circ },90^{\circ }\}$ on the proposed indoor recognition model using linear weighted fusion with $\beta =0^{\circ }$. Nonetheless, the proposed model still outperforms the baseline system, demonstrating the applicability of our approach. An error following a normal distribution with $\mu =0^{\circ }$ and $\sigma =120^{\circ }$ causes a drop in accuracy, resulting in the worst performance when compared to the baseline. However, such a high value of variance error is not practical in general.

### 5.5. Model Analysis and Partitioning for Inference

In a CNN model, each layer has its own set of learnable weights that are optimized during training by minimizing the classification loss. These parameters are typically saved in a model file that can be loaded into memory during inference. ONNX (Open Neural Network Exchange; GitHub repository: https://github.com/onnx/onnx, accessed on 26 January 2022.) is an important DL model format because it provides a common standard for representing DL models, making it easier to develop and deploy them across multiple frameworks and devices. We compared the model file size of each implementation (with MobileNet-v2) as shown in Table 4. The proposed computing strategy (composed of frozen layers and trained layers) is lighter than the four complete direction-driven CNNs by about 15.93 MB.

**Figure 7:**
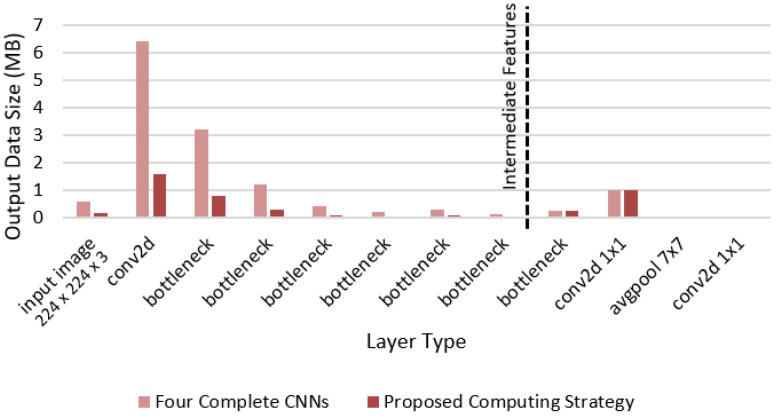
Per-layer output data size (MB) for four complete CNNs compared to the proposed computing strategy with MobileNet-v2. The dotted vertical line is the split point based on frozen layers as in Figure 4.

Additionally, Figure 7 describes the per-layer output data size (in MB) for the four complete CNNs represented in Figure 4a compared to the proposed computing strategies as in Figure 4b,c. We can observe the following. First, adopting the proposed computing strategy is better than using the four complete CNNs because the common frozen layers are implemented once rather than four times, resulting in less computation time and required power. Second, because the input image size is larger than the intermediate feature size, splitting the CNN into two parts (i.e., a first part running on the mobile device and a second part running on a cloud or edge server) may be more beneficial. As a result, the submodels are deployed in the manner described in Table 5 (i.e., partial offloading as in Figure 4c).

## 6. Conclusions and Future Work

In this paper, we propose a direction-driven multi-CNN indoor scene recognition system for room-level localization that uses embedded smartphone sensors to account for the camera heading direction if the user smartphone relative to magnetic north. We created our own dataset, which includes images with corresponding magnetic heading values. We also used and compared two heading-based weighted fusion techniques. Experiments showed that the proposed system outperforms the baseline system based solely on images. When dealing with indoor scene image data, the proposed system outperformed the traditional CNN image classification system. The proposed system relies on built-in smartphone sensors, which vary in quality and accuracy across different devices and environments and may have an impact on the overall performance of the system. We also investigated how magnetic heading error affected the proposed system, demonstrating the utility and stability of our method. Additionally, we discussed the current computing paradigms and how they apply to DL tasks. We also analyzed the effect of model partitioning on system computation during inference and proposed a hybrid computing strategy for our scene recognition system (i.e., model partial offloading between the mobile device and server). In the future, we will focus on the applicability of the proposed system in terms of maintenance, which has two components: first, when changes occur in previously studied rooms, affecting the performance of the system, and second, when additional rooms must be identified, requiring the system to be deployed over a larger area.

## Figures and Tables

**Table 1:** Comparison of the accuracy (${}_{avg}$(%)) between the baseline system and the proposed approach (best results are in bold).

Pretrained Model	Baseline	Proposed Approach			
		Linear Fusion			Cosinusoidal Fusion
		$\beta =0^{\circ }$	$\beta =30^{\circ }$	$\beta =45^{\circ }$	
SqueezeNet	67.52 ± 1.95	**81.02 ± 2.75**	77.50 ± 3.30	77.02 ± 3.30	79.52 ± 2.62
ShuffleNet	88.98 ± 2.03	**92.22 ± 0.41**	91.40 ± 0.54	91.34 ± 0.44	91.94 ± 0.69
MobileNet-v2	90.66 ± 1.80	**93.10 ± 0.56**	92.62 ± 1.03	92.50 ± 0.82	92.44 ± 0.82

**Table 2:** Comparison of the accuracy (${}_{avg}$(%)) between the baseline system and different selection rules (best results are in bold).

Pre-Trained Model	Baseline	Different Selection Rules		
		Opposite Quadrant Selection	One Random CNN	One Specific CNN
		with Linear Fusion ($\beta =0^{\circ }$)		
SqueezeNet	**67.52**	32.96	52.96	51.91
ShuffleNet	**88.98**	44.86	63.38	63.45
MobileNet-v2	**90.66**	46.30	63.92	64.21

**Table 3:** Comparison of the accuracy (${}_{avg}$(%)) between the baseline system and the proposed approach with linear fusion ($\beta =0^{\circ }$) simulating error on magnetic heading (best results are in bold and worst results are highlighted in red).

Pre-Trained Model	Baseline	Proposed Approach with Linear Fusion ($\beta =0^{\circ }$)				
		e=0	σ=30	σ=60	σ=90	σ=120
SqueezeNet	67.52	**81.02**	80.18	79.26	77.84	75.78
ShuffleNet	88.98	**92.22**	91.46	91.24	89.12	88.10
MobileNet-v2	90.66	**93.10**	92.86	92.60	91.30	89.60

**Table 4:** Model file size of the different implementations with MobileNet-v2.

Framework	Model	Model File Size
ONNX	Four complete CNNs	35 MB
	Proposed computing strategy	19.07 MB

**Table 5:** Proposed computing strategy submodel sizes and outputs with MobileNet-v2 based on partial offloading as in Figure 4c.

Submodels	Sub-Model File Size (MB)	Output Data Size (MB)	Computing Strategy
Frozen Layers	5.31	$3.136\times 10^{-2}$	On-device (user side)
A (Trained Layers)	$\}3.44\times 4$	$0.002\times 10^{-2}$	Cloud or edge server
B (Trained Layers)			
C (Trained Layers)			
D (Trained Layers)			

## Data Availability

Data are available upon reasonable request to the corresponding author.

## Abbreviations

List of mathematical symbols and acronyms:

**Symbol**	**Definition**
CNN	Convolutional neural network
DL	Deep learning
VPR	Visual place recognition
θ	Smartphone camera magnetic heading $\in [0^{\circ },360^{\circ }]$
*p*	Probability vector corresponding to the image inference output of a CNN
$\theta^{\prime }$	Modified smartphone camera magnetic heading $\in [0^{\circ },90^{\circ }]$
$\alpha (\theta^{\prime })$	Weighted parameter of the fusion method
β	Hyperparameter for piecewise linear weighted fusion
*e*	Magnetic heading error
ν	Mean
σ	Standard deviation
$\theta_{e}$	Smartphone camera magnetic heading subject to error $\in [0^{\circ },360^{\circ }]$
ONNX	Open Neural Network Exchange
